# Integrated transcriptomic and proteomic analysis reveals isolation and culture associated molecular changes in neonatal porcine pancreatic cell clusters

**DOI:** 10.1007/s11033-026-12376-8

**Published:** 2026-07-23

**Authors:** Chang-Yi Chen, Chen-Ling Chen, Chen-Wei Kao, Chen-Yi Chen, Wan-Wen Hung, Ching-Wen Chang, Jyuhn-Huarng Juang, Wan-Chun Li

**Affiliations:** 1 Institute of Oral Biology, College of Dentistry, National Yang Ming Chiao Tung University, Taipei, Taiwan; 2 Division of Endocrinology and Metabolism, Department of Internal Medicine and Center for Tissue Engineering, Chang Gung Memorial Hospital, Taoyuan, Taiwan; 3 Graduate Institute of Metabolism and Obesity Sciences, Taipei Medical University, Taipei, Taiwan; 4 TMU Research Center for Digestive Medicine, Taipei Medical University, Taipei, Taiwan; 5 Taipei Cancer Center, Taipei Medical University, Taipei, Taiwan; 6 Department of Medicine, College of Medicine, Chang Gung University, Taoyuan, Taiwan; 7 Department of Dentistry, College of Dentistry, National Yang Ming Chiao Tung University, Taipei, Taiwan; 8 Oral Medicine Innovation Center (OMIC), National Yang Ming Chiao Tung University, Taipei, Taiwan; 9 Department of Stomatology, Taipei Veterans General Hospital, Taipei, Taiwan

**Keywords:** Porcine Neonatal Pancreatic Cell Cluster, RNA sequencing, Proteomics, Culture adaptation, Extracellular matrix

## Abstract

**Background:**

While porcine Neonatal Pancreatic Cell Clusters (NPCCs) were potential alternative source of islets, their immaturity and heterogeneity make them inefficiently ameliorate hyperglycemia in rodents. Our previous work showed that NPCCs regress to a transitional-like state immediately after isolation and undergo dynamic transcriptional changes during short-term in vitro culture. The present study aimed to further delineate the molecular cues underlying these changes during porcine pancreatic tissue isolation and cultivation.

**Method:**

Transcriptomic and proteomic analysis were performed to compare Neonatal Porcine Pancreata (NPP), freshly isolated NPCCs (NPCCs-0D) and 3-day in vitro cultured NPCCs (NPCCs-3D). Using an in-house analysis pipeline, molecular differences between groups were identified.

**Results:**

NPCCs‑0D showed lower molecular abundance compared with NPP and NPCCs‑3D groups under the tested conditions. Gene Sets Enrichment Analysis (GSEA) revealed that pathways related to receptor signaling and cell–microenvironment interactions were enriched in NPP and NPCCs‑3D, highlighting the importance of external regulation in maintaining pancreatic tissue architecture. In contrast. organelle- and catalysis-associated gene sets were enriched in NPCCs‑0D, suggesting a transcriptionally active and adaptive state.

**Conclusion:**

Collectively, these findings indicate that molecular differences among NPP, NPCCs-0D, and NPCCs-3D reflect changes in tissue organization, cellular composition, and adaptation to isolation and culture conditions, rather than a linear differentiation process.

**Supplementary Information:**

The online version contains supplementary material available at 10.1007/s11033-026-12376-8.

## Introduction

With recent expedited advances in stem cell biology, gene editing and tissue engineering, β cell replacement therapy - primarily through transplantation - has been a successful proof-of-concept transformation toward achieving prolonged insulin independence in patients with Type 1 Diabetes Mellitus (T1DM) [1, 2]. Nevertheless, various challenges remain, including complex isolation procedure, inconsistent islets quality, limited availability of immunotype-matched donors, and the need for repeated transplantation and long-term immunosuppression to maintain glycemic control [3, 4]. Furthermore, as most transplantation studies were conducted in rodents, direct translation to humans remains challenging due to the physiological variance and distinct immune responses between rodent and humans or non-human primates [5, 6].

Besides donor-derived human islets, considerable effort has been devoted to developing renewable sources of insulin-producing cells. These include products generated from human pluripotent stem cells (hPSCs) as well as pancreatic tissues obtained from other species for xenotransplantation applications [7]. While stem cell-derived approaches have advanced substantially and entered clinical evaluation, challenges related to differentiation efficiency, long-term stability, and safety remain [8]. By contrast, porcine pancreatic tissues have been investigated for decades and continue to represent a practical source of transplantable endocrine tissue because of their biological similarity to the human pancreas and the availability of donor animals [9–11].

Among porcine pancreatic graft sources, neonatal preparations offer several practical advantages [10]. In comparison with adult islets, neonatal pancreatic cell clusters can be isolated more consistently and tolerate the isolation procedure relatively well [10, 12]. Although these cell clusters are capable of producing insulin and improving glucose control after transplantation, they generally require an extended post-transplant period before achieving functional competence [12, 13]. Consequently, short-term culture studies are more suitable for investigating immediate cellular responses to tissue dissociation and environmental adaptation than for evaluating complete endocrine maturation.

By taking advantage of immunofluorescence staining assays in isolated NPCC cells and transplants, our previous findings revealed that the percentage of PDX1^+^/Ins^−^ and Sox9^+^ pancreatic precursor-like cells significantly increased immediately after isolation before culture, termed as NPCCs-0D, compared with neonatal porcine pancreata. The transitional cell populations remain prominent over 4 days in culture; while endocrine differentiation, based on the detection of insulin^+^, glucagon^+^ and PP^+^ (Pancreatic Polypeptide) cells, increased in cultivated NPCC over time [14]. To better characterize the molecular consequences of pancreatic tissue dissociation and subsequent culture adaptation, we compared neonatal porcine pancreas (Neonatal Porcine Pancreata, NPPs), freshly isolated NPCCs (NPCCs-0D), and NPCCs maintained in culture for 3 days (NPCCs-3D). Transcriptomic and proteomic approaches were integrated to identify condition-dependent molecular signatures associated with tissue architecture, cellular heterogeneity, and environmental adaptation. Rather than viewing these experimental groups as successive developmental stages, we considered them distinct biological states generated by isolation and culture procedures.

The results indicate that a greater number of gene sets were detected in NPP and NPCCs‑3D groups compared with NPCCs-0D cells, whereas the gene sets associated with cellular organelles and catalysis activity are abundant in NPCCs-0D group. The loss of “niche” related cues such as the genes/proteins relevant to the cell-extracellular matrix (ECM) interaction, may contribute to the disruption of tissue organization and the induction of dynamic transcriptional responses following NPCC isolation, rather than directly determining a fixed cellular identity.

## Materials and methods

### Preparation and culture of NPCCs

All animal procedures (IACUC number: 2019091802) were conducted following a previously described procedure [10, 14] in accordance with the Institutional Animal Care and Use Committee (IACUC), Chang Gung Memorial Hospital (CGMH), Taoyuan, Taiwan. Pancreata from 1‑day‑old piglets of both sexes were minced into ~ 1–3 mm fragments and subjected to enzymatic digestion using collagenase type XI (Sigma-Aldrich, Cat No C7657) at 37 °C for 20 min in a controlled water bath. The resulting suspension was sequentially filtered through a nylon screen (500 𝛍m) [10] and washed in washing solution [Hanks′ Balanced Salt Solution (HBSS, Sigma-Aldrich, Cat No H9394) + 0.5% (w/v) Bovine Serum Albumin (BSA, Sigma-Aldrich, Cat No A2153)], centrifuged at 13,000 xg for 90 s, then cultured in RPMI medium (Sigma-Aldrich, Cat No R6504) supplemented with 50 mM (3-Isobutyl-1-methylxanthine, IBMX) (Sigma-Aldrich, Cat No I5879), 0.5% BSA, 2 mM L‑glutamine (Sigma-Aldrich, Cat No G7513), 10 mM nicotinamide (Sigma-Aldrich, Cat No N0636), and standard antibiotics (100 U/ml penicillin, 100 µg/ml streptomycin) (Sigma-Aldrich, Cat No P4333). Cultures were maintained at 37 °C in humidified 5% CO₂/95% air for three days to generate NPCCs‑3D. The medium was changed every 2 days.

### Quantitative RT-PCR (qPCR) analysis

Total RNA was extracted from NPP, NPCCs‑0D, and NPCCs‑3D using Invitrogen TRIzol reagent (Thermo Fisher Scientific Inc., Cat No 15596026). RNA concentrations were measured with a NanoDrop TM 1000 spectrophotometer, and cDNA was synthesized using the SuperScript III First‑Strand kit (Invitrogen, Cat No 18080051) following manufacturer’s protocol. The qPCR reactions were prepared with KAPA SYBR FAST ABI PRISM Master Mix and gene‑specific primers (Supplementary Table 1) [14], and run on a StepOnePlus™ Real‑Time PCR System (Applied Biosystems) at Instrumentation Resource Center, Yang Ming Campus, National Yang Ming Chiao Tung University. As 3 conditions without a defined “control” or “treated” group were compared, qPCR data were normalized using the ΔCt method, in which gene expression levels were normalized to an internal control gene, rather than being presented as ΔΔCt fold changes relative to a specific reference group. We selected the ΔCt method to reduce potential inter-experimental bias and to enable direct comparison of relative gene expression levels across samples. The internal control gene used for normalization is Ribosomal Protein Large Subunit 32 (RPL32) [14]. The qPCR analysis was performed on RNA samples prepared independently from the RNA-seq experiments.

### RNA-sequencing and analysis

RNA samples from NPP (*n* = 2), NPCCs‑0D (*n* = 2), and NPCCs‑3D (*n* = 3) were processed at the Genomic Medicine Core Laboratory, CGMH. Total RNA was extracted using TRIzol combined with BCP reagent (Invitrogen). RNA integrity (RIN) and concentration were assessed with an Agilent 2100 Bioanalyzer (RNA 6000 Nano kit). Library yield and fragment size distribution were measured using a Qubit^®^ 2.0 Fluorometer (dsDNA High Sensitivity kit) and Agilent DNA 1000 assay. GC content and quality value (QV) distributions confirmed normal profiles, with QV > 20 across all samples, corresponding to error rates below 1% (Supplementary Fig. 1).

Libraries were prepared using Universal Plus mRNA Seq with NuQuant (TECAN) and sequenced on an Illumina MiSeq platform (2 × 151 bp paired‑end). Raw reads were processed with CLC Genomics Workbench v10 (Tri‑I Biotech, Inc.) in combination with a custom Python-based pipeline designed for automated workflow management and reproducibility. Initial quality control was performed using FastQC (v0.12.0). Adapter trimming and removal of low-quality bases were conducted using Cutadapt (v5.0) with a quality threshold of Phred score ≥ 20 and minimum read length filtering [15] (Supplementary Fig. 2A). After preprocessing, > 99.8% of reads were retained across all samples, indicating high sequencing quality and minimal technical artifacts. Clean reads were aligned to the Sus scrofa reference genome (Sscrofa11.1) using STAR (v2.7.11b) [16] with default parameters optimized for splice-aware alignment in mammalian transcriptomes. Only uniquely mapped reads were retained for downstream analyses.

Gene expression was quantified as FPKM [17] values. In parallel, raw read counts were also generated to ensure compatibility with count-based differential expression methods. Across samples, between 18,882 and 21,275 genes were detected (Supplementary Fig. 2B), consistent with expected transcriptome complexity in porcine tissues. More than 75% of mapped reads were assigned to annotated exonic regions in most samples (except NPP‑2; Supplementary Fig. 2C), supporting high RNA integrity and accurate genome annotation. Overall, the observed mapping efficiency and gene detection rates are comparable to previously reported high-quality RNA-seq datasets [18], supporting the robustness and reliability of the analytical pipeline.

Differentially expressed genes (DEGs) were identified using DESeq2 (v1.46.0) [19] using thresholds of fold change > 2 and *p* ≤ 0.05. and GseaVis (v0.1.0). Pearson correlation statistics were applied to evaluate intra‑sample reproducibility. Principal Component Analysis (PCA) and Non‑metric Multidimensional Scaling (NMDS) were performed in R (prcomp function), and 3D PCA plots were generated using Cubemaker. Heatmaps of DEGs (FC > 2, *p* ≤ 0.05) were visualized with TreeView (v1.1.6r4). Functional enrichment was examined with Gene Ontology (GO), KEGG pathway analysis, and Gene Set Enrichment Analysis (GSEA) using clusterProfiler [20, 21].

### Qualitative proteomics analyses

Protein extracts from NPP, NPCCs‑0D, and NPCCs‑3D were prepared in detergent‑based lysis buffer (4% SDS in 20 mM HEPES, pH 7.5-8.0). Five microliter of protein lysates/sample are separated by electrophoresis and stained for SYPRO Ruby Protein Gel Stain (Molecular Probes/Invitrogen). Staining procedures were followed according to the manufacturer’s instruction [22]. In brief, after electrophoresis, gels were placed in a fixing solution (mixture of 50% methanol and 7% acetic acid) overnight in preparation for protein visualization. The ProXPRESS™ 2D Proteomic Image System (PerkinElmer Life and Analytical Sciences) was used to scan the SYPRO Ruby stained gels. A hundred microgram proteins from different samples were reduced with dithiothreitol, alkylated with iodoacetamide, and digested using sequencing‑grade trypsin (Promega, Madison, WI) following the filter‑aided sample preparation method. Peptides were acidified with trifluoroacetic acid, desalted using Sep‑Pak C18 cartridges (Waters Associates, Milford Mass, USA), and labeled with TMT 6‑plex reagents (Thermo Fisher Scientific) according to the manufacturer’s instructions. Labeled peptides were pooled, fractionated under high‑pH reversed‑phase conditions (Pierce High pH Fractionation Kit, Thermo Fisher Scientific), and re‑suspended in 0.1% formic acid for LC‑MS/MS analysis. Mass spectrometry was performed on a Q Exactive™ HF instrument (Thermo Fisher Scientific) coupled to an UltiMate™ 3000 RSLCnano HPLC system. Peptides were separated on a 50‑cm EASY‑Spray™ C18 column using a gradient of acetonitrile at 250 nL/min over ~ 165 min. Spectra were acquired in positive ion mode with data‑dependent acquisition. The top 15 precursor ions (375–1400 m/z) were fragmented in high‑collision dissociation (HCD) mode with normalized collision energy of 33 ± 1. Full MS scans were acquired at 60,000 resolution (m/z 200), AGC target 3e6, and maximum injection time 50 ms; MS/MS scans were acquired at 15,000 resolution, AGC target 5e4, and maximum injection time 100 ms. Dynamic exclusion was set to 20 s.

Raw data were processed in Proteome Discoverer (Thermo Fisher Scientific) and searched with Mascot and Sequest HT against the Sus scrofa UniProt reference proteome (UP000008227, 23,223 entries). Search parameters included carbamidomethylation of cysteine as a fixed modification, and oxidation (Met), pyro‑Glu formation (N‑terminus), acetylation (protein N‑terminus), and TMT labeling (peptide N‑terminus and Lys) as variable modifications. A maximum of two missed cleavages was allowed, with MS tolerance of 10 ppm and MS/MS tolerance of 0.02 Da. Peptide and protein identifications were accepted at false discovery rate (FDR) < 1%. Proteins were considered valid if supported by ≥ 2 unique peptides with ≥ 2 peptide spectrum matches. Due to limitations in the availability of quantitative intensity data, proteomic analysis in this study is presented as qualitative identification of proteins rather than quantitative comparison. Proteins identified with high confidence (based on unique peptides and sequence coverage) were used for downstream functional and pathway-level interpretation. Protein accession IDs obtained from LC–MS/MS analysis were used as unique protein identifiers throughout the study.

The number of unique peptides assigned to each protein accession was obtained from the processed datasets. Only peptides uniquely mapping to a single protein were included to ensure specificity of protein identification. Protein sequence lengths, defined as the total number of amino acids per protein, were retrieved from the UniProt database using a list of 1,699 accession IDs and compiled into a separate worksheet. Estimated protein sequence coverage was calculated by combining the number of unique peptides with the corresponding protein length. As individual peptide sequences and their exact lengths were not summed, coverage was approximated using an average tryptic peptide length. The values were calculated based on peptide lengths of 10 amino acids, representing average estimates. Coverage for each protein was calculated according to the following formula:

$$\begin{aligned}&Coverage \:(\%) = (Number\: of \:unique \:peptides\\&\quad \times peptide\: length(aa))/protein \:length(aa) \times 100\end{aligned}$$

Proteins with no detected unique peptides were assigned a coverage of 0%. Coverage calculations were performed separately for each experimental condition (e.g., NPP, NPCC-0D, NPCC-3D), and mean coverage values were subsequently calculated across replicates to obtain representative estimates for each group. The final Excel worksheet contained protein accession numbers, protein lengths, unique peptide counts, and calculated coverage values, allowing for systematic comparison of protein detection and sequence representation across experimental conditions. The accession IDs with (1) 0% coverage in any condition and (2) missing values of any abovementioned index were excluded for further analysis. A total of 512 accession IDs were included for final analysis.

### Statistical analysis

All data are expressed as mean ± standard error of the mean (SEM). Comparisons between two groups were performed using unpaired Student’s t‑tests, with statistical significance defined as *p* < 0.05.

## Results

**Fig. 1:**
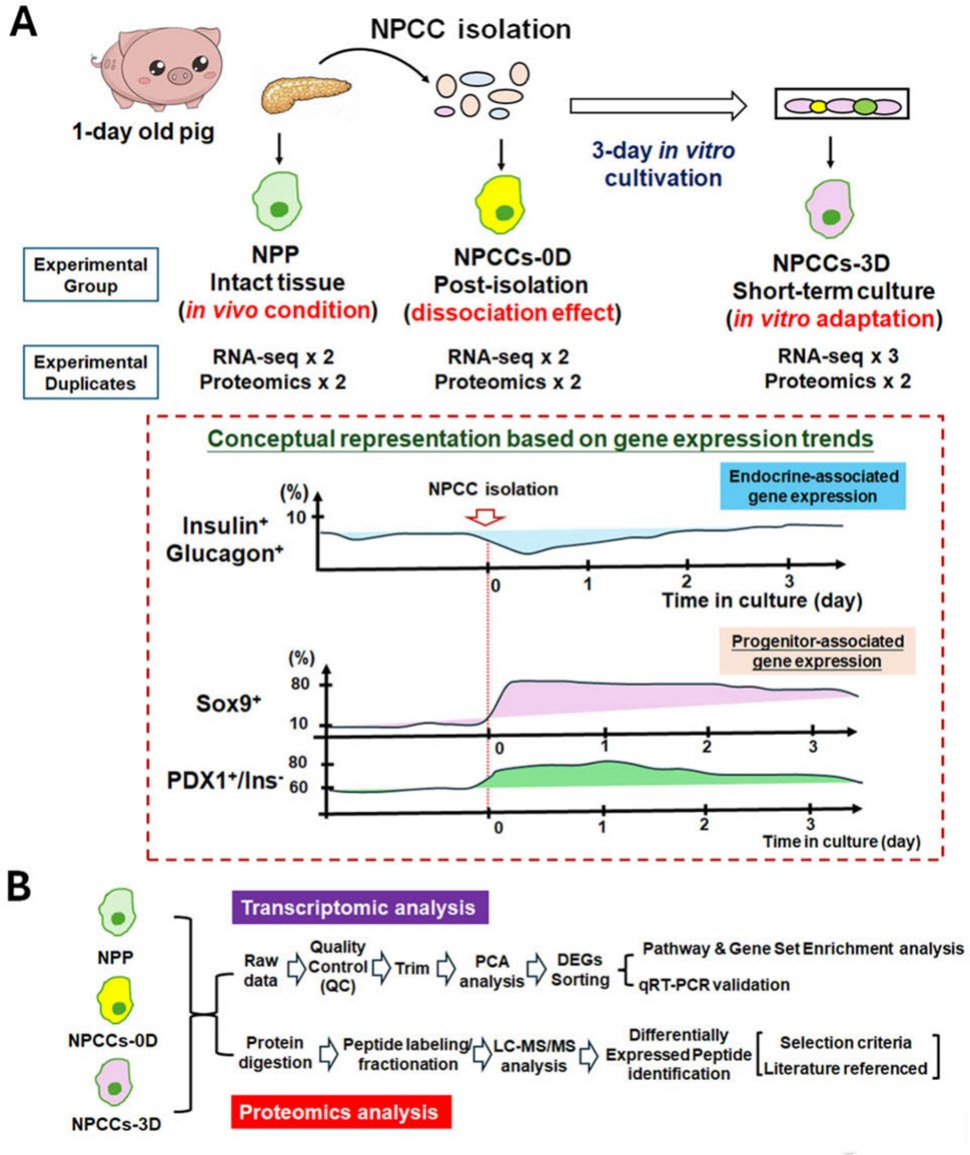
Experimental design and analytical workflow for multi-omics profiling of neonatal porcine pancreatic tissues and derived cell clusters. (**A**) Schematic overview of sample preparation. Pancreata from 1‑day‑old pigs were used to represent intact tissue under in vivo conditions (Neonatal Porcine Pancreata; NPP). Neonatal porcine pancreatic cell clusters immediately after enzymatic isolation (NPCC‑0D) reflect tissue dissociation and epithelial enrichment, whereas NPCCs cultured for 3 days in vitro (NPCC‑3D) represent short‑term culture adaptation. Numbers of biological replicates used for transcriptomic and proteomic analyses are indicated for each condition. The dotted box illustrates a conceptual representation of dynamic changes in endocrine‑associated and progenitor‑associated gene expression following isolation and short‑term culture, based on Li et al., 2018. (**B**) Analytical workflow for transcriptomic and proteomic profiling, including RNA sequencing (quality control, trimming, PCA, differential gene expression, pathway enrichment, and qRT‑PCR validation) and proteomics analysis (protein digestion, peptide labeling/fractionation, LC‑MS/MS, and protein identification)

To investigate molecular differences associated with tissue dissociation and short-term culture, three neonatal porcine pancreatic conditions were analyzed (Fig. 1). Intact NPP represents the in vivo tissue condition, while freshly isolated neonatal porcine pancreatic cell clusters (NPCC‑0D) reflect the immediate effects of enzymatic dissociation and loss of tissue architecture. NPCCs cultured for three days (NPCC‑3D) represent short-term adaptation to in vitro conditions. We interpret the experimental groups represent different biological contexts, but not sequential stages of differentiation. Samples were subjected to integrated transcriptomic and proteomic analyses to assess global molecular changes across conditions. Marker gene expression patterns associated with endocrine and transitional features were also examined to provide context for observed transcriptional differences. Importantly, these comparisons are intended to highlight condition-dependent molecular variation rather than a strict developmental hierarchy.

### Expression patterns of functionally categorized genes reveal dynamic and adaptive transcriptional cues during NPCC isolation and cultivation

**Fig. 2:**
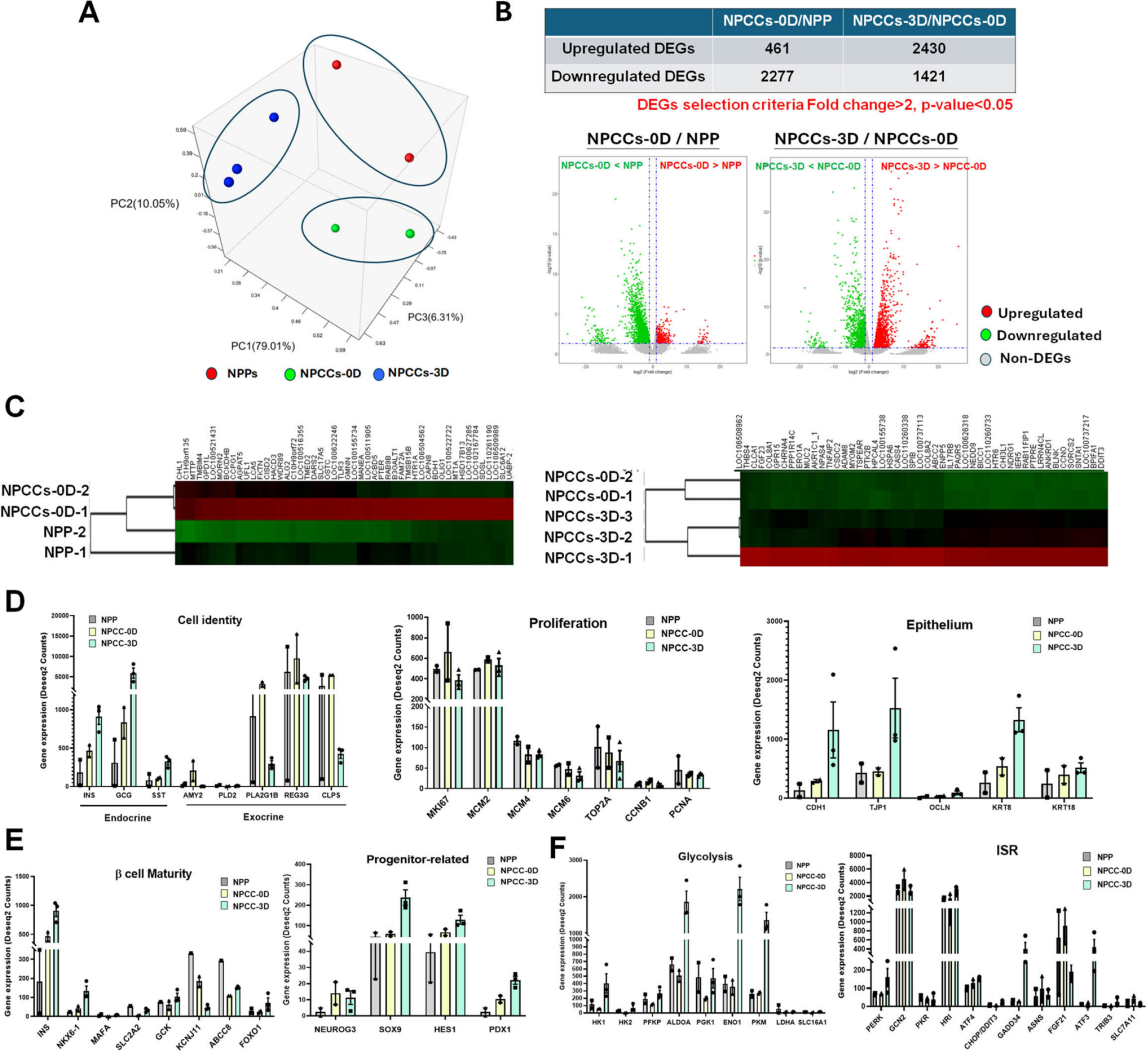
Global transcriptomic variation and functional gene expression patterns across NPP, NPCC‑0D, and NPCC‑3D conditions. (**A**) P Principal component analysis (PCA) of RNA-seq data showing transcriptional variation among NPP (red), NPCC-0D (green), and NPCC-3D (blue). Each point represents an individual biological replicate, with clustering reflecting condition-dependent transcriptional profiles. (**B**) DEGs analysis between sample groups. Upper panel summarized significantly upregulated and downregulated genes in each comparison (NPCC‑0D vs. NPP and NPCC‑3D vs. NPCC‑0D; fold change > 2, *p* < 0.05). Lower panels showed the volcano plots illustrating the distribution of gene expression distributions; upregulated genes are shown in red, downregulated genes in green, and non-significant genes in gray. (**C**) Heatmap showing hierarchical clustering of the top DEGs for each comparison (left: NPCC‑0D vs. NPP; right: NPCC‑3D vs. NPCC‑0D). Rows represent genes and columns represent biological replicates, illustrating condition-specific expression patterns and inter-replicate variability. (**D–F**) Expression profiles of functionally categorized genes derived from RNA‑seq datasets (DESeq2 counts). (**D**) Cell identity (endocrine and exocrine markers), proliferation-associated genes, and epithelial markers. (**E**) β-cell maturity–associated genes and pancreatic progenitor-related transcription factors. (**F**) Metabolic pathway genes (glycolysis) and integrated stress response (ISR) genes. Data are presented as gene expression counts across NPP, NPCC‑0D, and NPCC‑3D conditions, highlighting heterogeneous and adaptive transcriptional changes associated with tissue dissociation and short-term culture

For RNA-seq analysis, Principal Cluster Analysis (PCA) confirmed that NPP, NPCCs-0D and NPCCs-3D exhibit distinct genetic profiles (Fig. 2A) and basic information including number of reads, total mapped read, number of detected genes and exonic and intronic mapped reads are detailed in Supplementary Fig. 2. These results from quality control assays warrant further investigations of current set of RNA-seq data. The parameters of fold change > 2 and p-values < 0.05 were further set as a cut-off value to define Differentially Expressed Genes (DEGs) among groups (Supplementary Table 2). The results showed that 461 DEGs are upregulated and 2277 DEGs are downregulated in NPCCs-0D compared with NPPs; while 2430 DEGs are upregulated and 1421 DEGs are downregulated in NPCCs-3D compared with NPCCs-0D (Fig. 2B). By conversion of FPKM to Z-score, the heatmaps of comparisons between various groups clearly separated NPP, NPCCs-0D and NPCCs-3D groups (Fig. 2C and Supplementary Fig. 3).

To further interpret the biological significance of transcriptomic differences, certain candidate genes were grouped into functionally relevant categories, including exocrine/endocrine genes, proliferation-related genes, EMT markers, pancreatic progenitor markers, mature β-cell associated genes, metabolic pathways, and integrated stress response (ISR) genes (Fig. 2D and F and Supplementary Table 3). For basic tissue/cell characteristics (Fig. 2D), simultaneous detection of exocrine- and endocrine-related transcripts in NPCCs‑0D supports the presence of a mixed cellular population with features of an intermediate adaptive state, rather than a uniform or fully differentiated cellular state [23]. The continuous increasing expression of endocrine markers insulin, glucagon and somatostatin, accompany with a drop of most exocrine genes, supported the previous findings showing that in vitro NPCC cultivation enriched endocrine populations [10, 14]. In addition to endocrine associated genes, NPCCs-3D group also showed a greater expression for epithelium-related genes, implying potential epithelial adaptive activity to enzymatic dispersion during NPCC isolation, alike previous reported in vitro self-assembling mouse pancreatic organoid [24]. In addition, genes related to cell proliferation, including PCNA and members of the minichromosome maintenance (MCM) complex, were detected across NPCC samples, indicating the presence of cells engaged in the cell cycle. Notably, this result should be interpreted this result in a more cautious way since proliferation markers such as Ki‑67 and PCNA are widely recognized as indicators of cell cycle activity rather than definitive markers of cell identity or differentiation state [25, 26].

Genes associated with pancreatic progenitor features, including SOX9, HES1, Neurogenin 3 (Neurog3) and PDX1, were detected across all conditions with variable expression pattern. SOX9 and related transcription factors are known to play key roles in maintaining pancreatic progenitor populations and regulating epithelial plasticity during development [23, 27, 28]. Interestingly, at the same time, β-cell maturity associated genes, including INS, NKX6‑1, GCK, and ABCC8, were also expressed, although with differing levels across conditions (Fig. 2E). In a physiological level, metabolism-related genes, particularly those involved in glycolysis (e.g., HK1, PFKP, PKM, and ENO1), were also upregulated in NPCC‑3D samples. These findings are compatible with metabolic adaptation processes through which cells reorganize energy utilization in response to altered environmental and biosynthetic requirements [29, 30]. Similarly, increased expression of ISR-related genes, including PERK, GCN2, ATF4, GADD34, CHOP and FGF21, indicates activation of stress response pathways (Fig. 2F). The ATF4–HSPA5 axis is a well-established component of the endoplasmic reticulum stress response, which plays a central role in maintaining cellular homeostasis under adverse conditions such as metabolic imbalance and environmental stress [31, 32]. Altogether, these gene category–based observations are consistent with pathway-level analyses and support the interpretation that NPCC samples undergo complex transcriptional adaptation to isolation and culture conditions, characterized by concurrent modulation of structural, lineage-associated, metabolic, and stress-response genes. These combined features likely reflect the influence of tissue dissociation, altered cellular composition, and short-term culture adaptation.

**Fig. 3:**
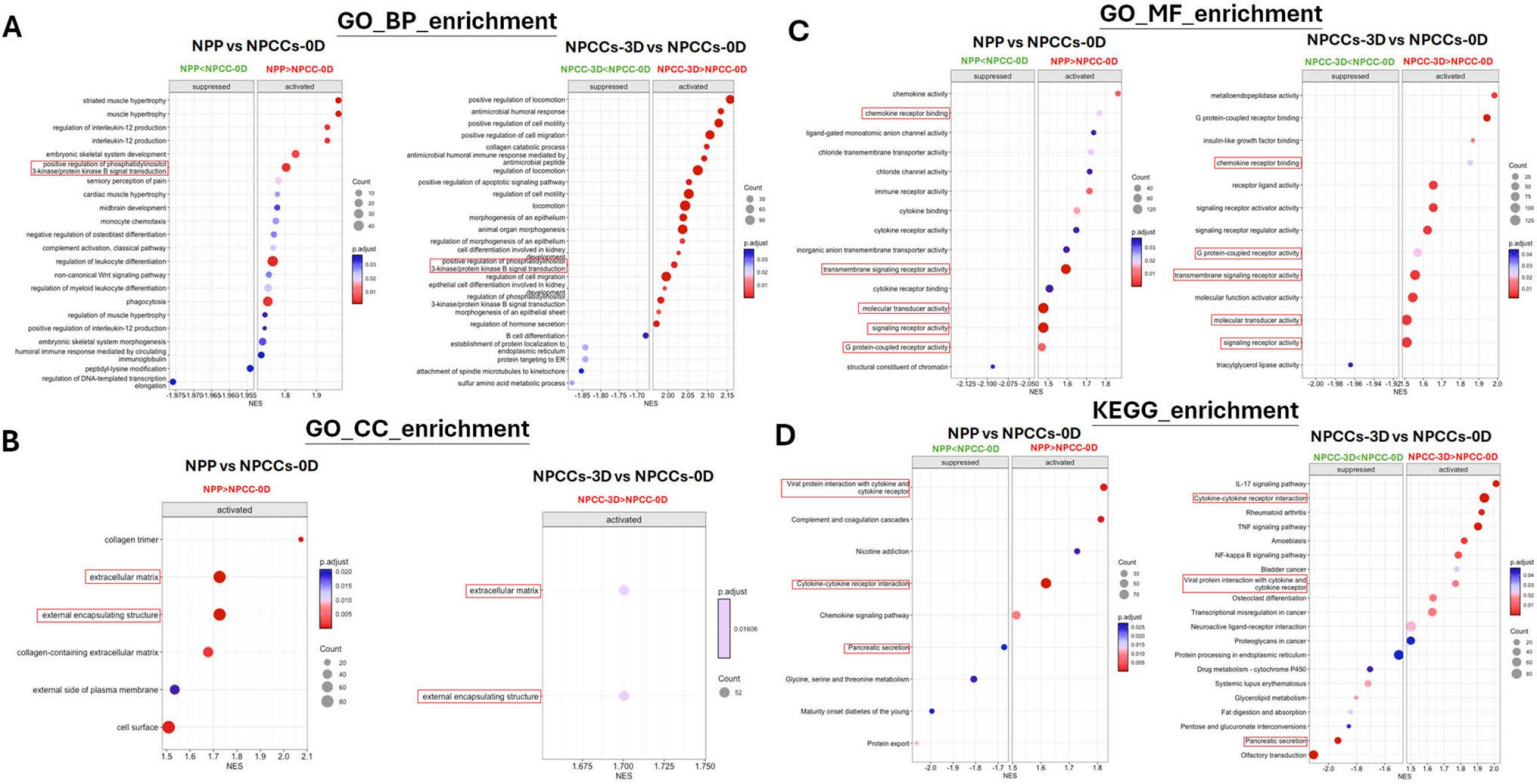
Functional enrichment analysis reveals extracellular matrix remodeling and signaling pathway alterations across experimental conditions. Gene Ontology (GO) and Kyoto Encyclopedia of Genes and Genomes (KEGG) enrichment analyses were performed for differentially expressed genes from NPP vs. NPCC‑0D and NPCC‑3D vs. NPCC‑0D comparisons. (**A**) GO Biological Process (BP) enrichment. (**B**) GO Cellular Component (CC) enrichment. (**C**) GO Molecular Function (MF) enrichment. (**D**) KEGG pathway enrichment analysis. For all panels, dot size represents the number of genes assigned to each pathway, and color indicates adjusted p-value. The x-axis represents normalized enrichment score (NES). Comparisons are separated into negatively enriched (suppressed) and positively enriched (activated) gene sets relative to NPCC‑0D. Red boxes highlight representative pathways shared between comparisons, including extracellular matrix–related processes and receptor-mediated signaling pathways

To further categorize function and related pathways of DEGs, GO (Gene Ontology, for gene function classification) and KEGG (Kyoto Encyclopedia of Genes and Genomes, for pathway classification) analyses were performed [33]. DEGs selected from comparisons of NPPs/NPCCs-0D and NPCCs-3D/NPCCs-0D were classified into three GO categories including Biological Process (BP, Fig. 3A), Molecular Function (MF, Fig. 3B), and Cellular Component (CC, Fig. 3C) as well as in KEGG database (Fig. 3D). The Normalized Enrichment Score (NES), a metric used to quantify the degree to which DEGs are enriched within a particular biological pathway or function, was created for analysis. The results showed that a greater number of gene sets were enriched in more differentiated NPPs and NPCCs-3D groups. Certain key gene sets including PI3K-PKB signaling pathway, extracellular matrix, chemokine receptor, G-protein receptor, transmembrane signaling receptor and cytokine-cytokine receptor interaction are significantly enriched in NPPs and NPCCs-3D groups (circled by red box in Fig. 3), suggesting that homeostatic integrity of cell-microenvironment interactions and receptor mediated signaling activity could be crucial for adaptative maintenance.

### Transcriptomic shifts highlight ECM remodeling and organelle downregulation during state determination in NPCCs

**Fig. 4:**
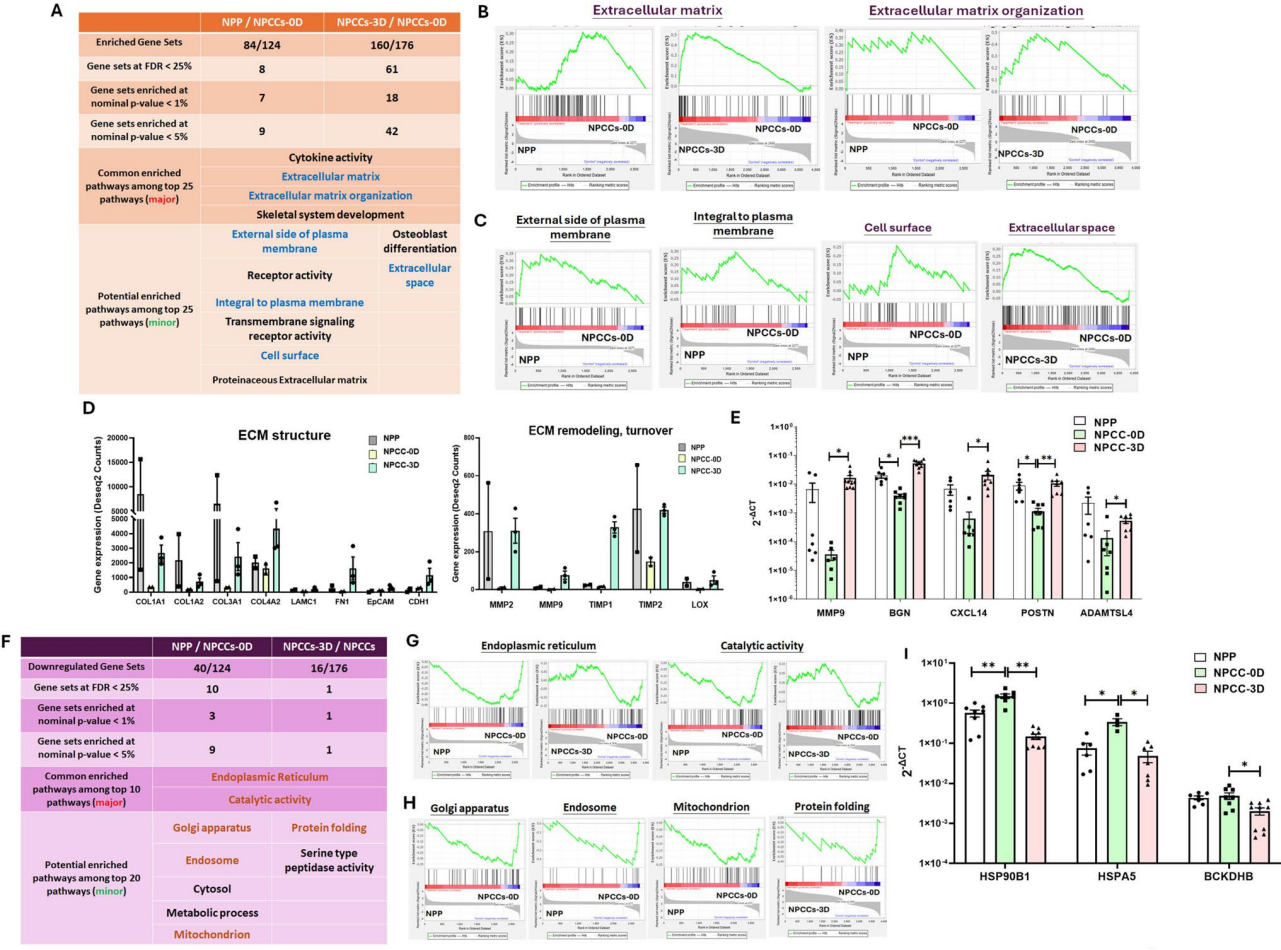
Gene set enrichment and validation analyses reveal extracellular matrix–associated and metabolic pathway alterations across NPCC conditions. (**A**) Summary of Gene Set Enrichment Analysis (GSEA) results for NPP vs. NPCC‑0D and NPCC‑3D vs. NPCC‑0D comparisons, including numbers of enriched gene sets under different statistical thresholds and commonly enriched pathways. (**B**) Representative GSEA enrichment plots for major pathways associated with ECM and ECM organization, showing enrichment trends across comparisons. (**C**) Representative GSEA plots for gene sets related to cell surface structure and membrane-associated components, including external side of plasma membrane, integral to plasma membrane, cell surface, and extracellular space. (**D**) RNA‑seq–derived expression profiles (DESeq2 counts) of ECM structural genes (e.g., collagens, laminins, fibronectin) and ECM remodeling/turnover genes (e.g., MMPs, TIMPs, LOX) across NPP, NPCC‑0D, and NPCC‑3D conditions. (**E**) Quantitative RT‑PCR (qRT‑PCR) analysis of selected ECM-related genes (MMP9, BGN, CXCL14, POSTN, ADAMTSL4), showing relative expression levels across experimental groups. Data are presented as 2^-ΔCt values with statistical comparisons. Data are presented as Mean ± SEM (N$\:\ge\:$3). ***p < 0.001. **p < 0.01; *p < 0.05. (**F**) Summary of downregulated gene sets identified by GSEA, including statistical thresholds and commonly enriched pathways (major and minor categories). (**G**) Representative GSEA enrichment plots for major downregulated pathways, including endoplasmic reticulum–associated and catalytic activity gene sets. (**H**) GSEA plots for selected minor pathways related to intracellular organelles and protein processing (e.g., Golgi apparatus, endosome, mitochondrion, and protein folding). (**I**) qRT‑PCR validation of representative genes associated with endoplasmic reticulum function and catalytic activity (HSP90B1, HSPA5, BCKDHB). Data are presented as 2^-ΔCt values with statistical comparisons. Expression levels were normalized to the internal control gene RPL32 using the ΔCt method. Values are presented as relative expression levels across groups

To determine whether the predefined gene sets show statistically significant, concordant differences between groups, Gene Set Enrichment Analysis (GSEA) were applied. The results found that 84 out of 124 gene sets and 160 out of 176 gene sets are enriched in more differentiated NPP and NPCCs-3D group compared with transitional NPCCs-0D cells, respectively. Among them, the “major” pathways which are gene sets significantly altered in both NPP/NPCCs-0D and NPCCs-3D/NPCCs-0D groups and “minor” pathways which are gene sets significantly altered either in NPP/NPCCs-0D or NPCCs-3D/NPCCs-0D group are defined (Fig. 4A). In agreement with GO/KEGG analysis, gene sets related to Extracellular matrix and Extracellular matrix organization were significantly abundant in NPP and NPCCs-3D compared with NPCCs-0D group (Fig. 4B). Furthermore, gene sets related to cell structural integrity such as gene sets relevant to external side of plasma membrane, integral to plasma membrane and cell surface (enriched in NPP/NPCCs-0D) as well as extracellular space (enriched in NPCCs-3D/NPCCs-0D) could potentially contribute to cell adaptative capacity (Fig. 4C). Previous studies have demonstrated that the pancreatic microenvironment is highly dependent on ECM composition, with collagens and fibronectin playing essential roles in maintaining tissue organization and regulating cell behavior [34, 35]. Consistent with enrichment analyses, genes associated with ECM structure and remodeling, such as COL1A1, COL3A1, and FN1, showed increased expression in NPCC‑3D compared with NPCC‑0D, indicating enhanced matrix-associated transcriptional activity. These ECM-related signatures are consistent with changes in cell–matrix interactions following tissue dissociation and short-term culture (Fig. 4D). Quantitative RT-PCR (qRT-PCR) analysis to examine consistency of transcriptomic trends of RNA-seq uncovered DEGs in newly isolated specimens was performed. Adaptation promoters including Matrix MetalloProteinase-9 precursor (MMP9), Biglycan (BGN) (GO_CC_Extracellular Matrix gene set), C-X-C motif chemokine 14 precursor (CXCL14) (GO_MF_Cytokine Activity gene set), Periostin (POSTN) and ADAMTS-like protein 4 (ADAMTSL4) (GO_BP _Extracellular Matrix Organization gene set) showed expression trends consistent with RNA-seq analysis, providing supportive evidence for the observed transcriptional patterns (Fig. 4E).

On the contrary, it was found that 40 out of 124 gene sets and 16 out of 176 gene sets are downregulated in NPP and NPCCs-3D group compared with NPCCs-0D cells, respectively (Fig. 4F). Two major pathways including gene sets related to endoplasmic reticulum and catalytic activity were defined (Fig. 4G), whilst numbers of minor gene sets related to cellular organelles such as gene sets relevant to golgi apparatus, endosome and mitochondrion (reduced in NPP/NPCCs-0D) as well as protein folding and serine-type peptidase activity (reduced in NPCCs-3D/NPCCs-0D) were uncovered (Fig. 4H). qRT-PCR analysis also confirmed that stress sensor and catalysis associated factors including Endoplasmin precursor (HSP90B1), 78 kDa glucose-regulated protein (HSPA5) (GO_CC_Endoplasmic Reticulum gene set) and mitochondrial 2-oxoisovalerate dehydrogenase subunit beta (BCKDHB) (GO_MF_Catalytic Activity gene set) were abundantly expressed in NPCCs-0D group, in agreement with RNA-seq results (Fig. 4I).

### Proteomics analysis unveiled key translational cues for NPCC cell state

**Fig. 5:**
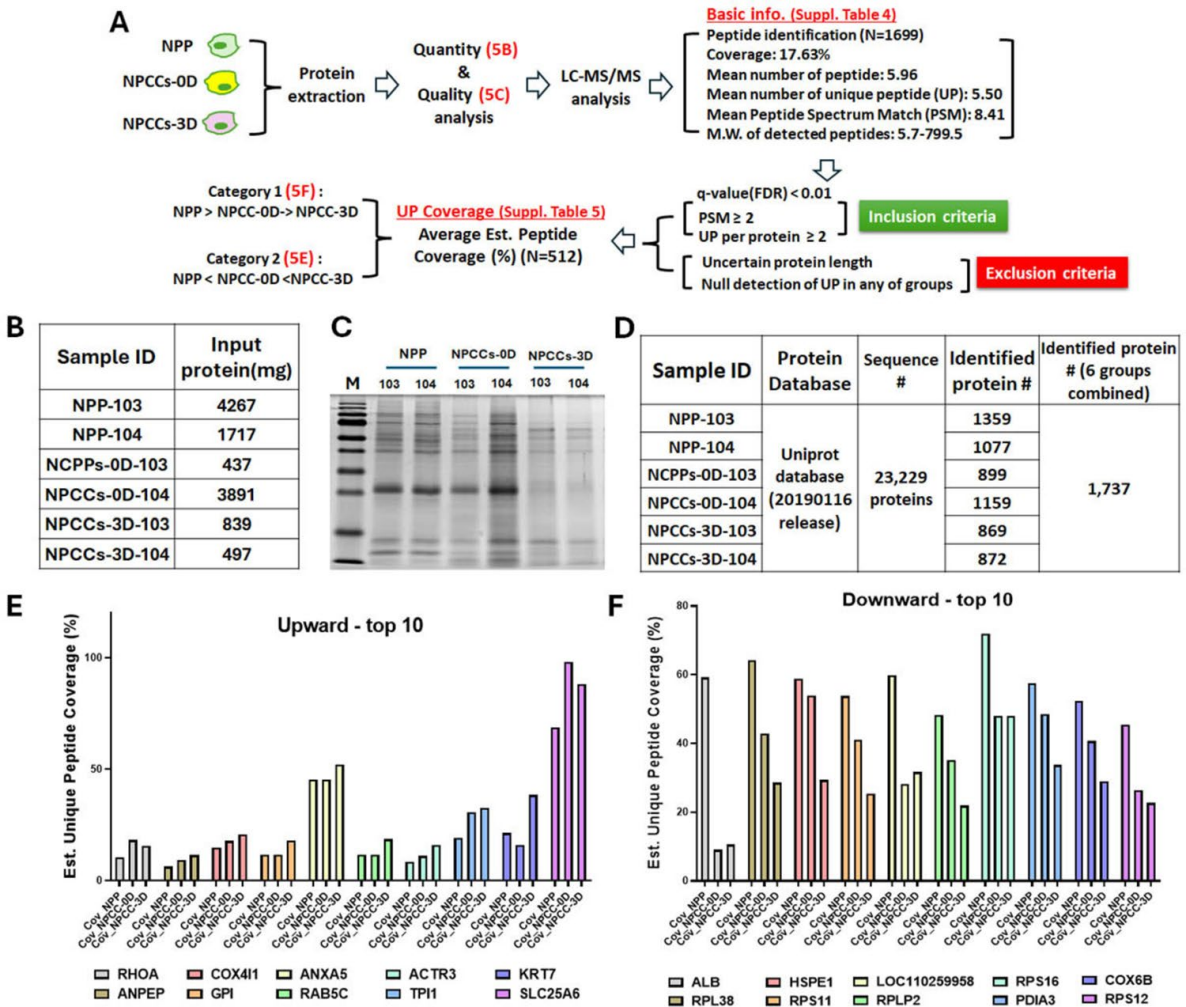
Proteomic profiling and peptide coverage analysis of neonatal porcine pancreatic samples. (**A**) Overview of the proteomics workflow, including protein extraction, quality and quantity assessment, LC–MS/MS analysis, peptide identification, and downstream filtering criteria. Inclusion criteria (false discovery rate < 1%, peptide spectrum match ≥ 2, and ≥ 2 unique peptides per protein) and exclusion criteria are indicated. Categories of proteins with increasing or decreasing estimated peptide coverage across conditions are defined. (**B**) Summary of total protein input amounts used for proteomic analysis across individual samples. (**C**) Representative SYPRO Ruby–stained gel showing overall protein quality and distribution for NPP, NPCC‑0D, and NPCC‑3D samples. (**D**) Summary of protein identification results based on UniProt database mapping, including numbers of identified proteins in each sample and combined dataset. (**E**) Top 10 proteins showing increased estimated peptide coverage across conditions (“upward” trend), presented as approximate sequence coverage (%) derived from unique peptide counts. (**F**) Top 10 proteins showing decreased estimated peptide coverage (“downward” trend) across conditions. Estimated peptide coverage values provide a qualitative indicator of protein representation across samples and do not reflect absolute protein abundance

In addition to transcriptomic analysis, molecular alterations in translational level were also examined in 2 sets of NPP/NPCCs-0D/NPCCs-3D specimens using a qualitative proteomics assay (Supplementary Table 4). The basic experimental information and selection criteria are listed in Fig. 5A. The total quantity ranged from 400 to 4000 mg proteins were extracted (Fig. 5B), and equal volume (5 µl) of protein lysates were separated in a 2-dimensional electrophoresis for quality analysis (Fig. 5C). LC‑MS/MS analysis revealed that identified peptides were mapped against 1,699 proteins among the reference proteome in Uniprot database of *Sus scrofa*. The general selection criteria were applied: (1) protein & peptide identifications False Determinate Rate (FDR, q-value) needs to be smaller than 0.01, (2) the Peptide Spectrum Match (PSM) of candidate peptides needs to be greater than 2, (3) the proteins identified with at least 2 unique peptides, and (4) rejection for Accession ID with uncertain protein length or null detection of unique peptide in any of experimental groups. Under the selection criteria, a total of 1,737 proteins across samples were identified by aligning with protein sequence database of *Sus scrofa*, with 512 proteins were analyzed to compare estimated sequence coverage across all 6 experimental conditions (Fig. 5D and Supplementary Table 5).

Further analysis indicated that the mean coverage decreased progressively from NPP (21.42%) to NPCCs-0D (18.81%) and NPCCs-3D (15.41%), indicating an overall reduction in peptide representation under NPCCs conditions. Comparative analysis revealed that only 21.1% (108/512) of proteins exhibited increased coverage in NPCCs-0D relative to NPP, while this proportion further dropped to 11.3% (58/512) in NPCCs-3D. Consistently, most proteins (> 80%) showed reduced coverage in NPCCs, particularly in condition of NPCCs-3D. To better determine the changing trend of UP coverage among conditions, the “virtual” slopes linking averaged estimated peptide coverage values between NPP (condition 1), NPCCs-0D (condition 2) and NPCCs-3D (condition 3) of all mapped proteins were determined. The top 10 upward and downward Accession IDs were defined (Fig. 5E and F). Despite the global trend of reduction of peptide coverage in NPCCs, a subset of proteins demonstrated marked increases in NPCC-3D, with the most coverage for proteins A0A287AVQ0 [ADP/ATP translocase 3 (SLC25A6); +19.61%], A0A287AG48 [Keratin 7 (KRT7]; +17.09%), and A0A286ZRV2 [Triosephosphate isomerase (TPI1); +13.46%]. In contrast, several proteins exhibited substantial reductions in coverage, including A0A287BAY9 [Serum Albumin (Alb); −48.56%] and F2Z568 [Ribosomal protein L38 (RPL38); −35.71%), indicating condition-specific loss of peptide detection. Notably, approximately 19.7% (101/512) of proteins showed increased coverage in NPCC-3D compared to NPCC-0D, suggesting that in vitro culture may potentially enhance detection of a specific subset of proteins despite the overall decrease in global coverage.

Proteomic analysis identified a broad range of proteins across NPP, NPCC‑0D, and NPCC‑3D samples. Although quantitative intensity-based measurements were not available, protein identification data, supported by peptide counts and sequence coverage, provided insight into the molecular composition of each condition. Across all samples, proteins associated with extracellular matrix organization (e.g., collagens and fibronectin), metabolic processes, and cellular signaling were consistently detected. The presence of these proteins corroborates transcriptomic pathway analysis and highlights the importance of extracellular and signaling components in pancreatic tissue and NPCC preparations. Taking individual condition into account, the NPCCs‑0D samples exhibited a wide range of identified proteins, reflecting a heterogeneous and dynamic cellular composition immediately following tissue dissociation. In addition, canonical exocrine proteins were detected across NPCC samples, supporting the presence of residual acinar components and reinforcing the heterogeneous nature of NPCC preparations. NPCCs‑3D samples retained proteins associated with extracellular structure and cellular communication, suggesting that short-term culture maintains key structural and signaling components. Importantly, protein identification patterns were generally consistent across biological replicates, as reflected by comparable peptide counts and sequence coverage (Supplementary Table 5), supporting the robustness of protein detection. In summary, proteomic data provides qualitative support for pathway-level observations derived from transcriptomic analysis, particularly in relation to extracellular matrix organization, signaling pathways, and cellular adaptation to isolation and culture conditions.

### Integration of transcriptomic and proteomic datasets

In agreement with previous studies [36, 37], integration of RNA-seq and proteomics data revealed both concordant and discordant molecular patterns across NPP, NPCC-0D, and NPCCs-3D conditions. Mapping of gene symbols to protein accession IDs using UniProt [38] demonstrated that a subset of molecules exhibited consistent regulation at both levels. Notably, the cytoskeletal gene KRT7 and its corresponding protein A0A287AG48 showed concordant upregulation, indicating structural remodeling under NPCCs-3D conditions. Similarly, GPI (P08059), a glycolytic enzyme with additional extracellular signaling functions, exhibited coordinated increases, supporting metabolic activation and adaptive signaling.

In contrast, prominent proliferation-associated genes, including MKI67 and MCM family members, displayed strong enrichment at the RNA level but lacked corresponding increases at the protein level, indicating discordant regulation. This discrepancy is consistent with well-established limitations in mRNA–protein correlation due to post-transcriptional and translational regulation, as well as technical constraints in proteomic detection. Additionally, proteins such as albumin (A0A287BAY9) showed marked reduction at the protein level without clear transcriptional changes, suggesting environmental or extraction-related effects.

Overall, these findings indicate that NPCC conditions induce robust transcriptional activation of proliferation pathways while selectively enriching structural and metabolic proteins at the proteome level, highlighting a multi-layered regulatory mechanism underlying three-dimensional cellular organization.

## Discussion

The present study examined global molecular alterations occurring during NPCC isolation and early culture adaptation. Comparison of neonatal pancreatic tissue, freshly isolated cell clusters, and short-term cultured NPCCs revealed substantial differences in transcriptional and proteomic profiles. These findings support the concept that tissue dissociation and subsequent culture exposure generate distinct molecular states that cannot be interpreted simply as sequential stages of differentiation. It is also noteworthy that endocrine markers are widely detected across conditions, with highest level in NPCCs-3D; in contrast, mRNA expression of exocrine transcripts enriched in NPCCs-0D and quickly dropped in 3-day cultivation, implying that endocrine identity is favorably enhanced over exocrine state during NPCC culture [14].

Current data suggested that NPCCs-D0 represent a transitional cell population that is influenced by enzymatic digestion and loss of native tissue structure. Accordingly, the observed expression of progenitor-related markers is more likely indicative of cellular flexibility and population heterogeneity than evidence of a discrete progenitor compartment. At first glance, this interpretation appears to contradict our earlier findings [14], where PDX1 and SOX9 expression increased after 1–2 days of culture. This discrepancy can be explained by methodological differences: the prior study relied on immunostaining of selected markers, while the present work employs bulk RNA-seq and proteomics to capture global molecular profiles. Moreover, the interpretation of proliferation markers also requires caution. Although genes such as *MKI67* and *PCNA* were expressed in NPCC samples, these markers broadly reflect cell-cycle activity rather than overall proliferative potential or developmental status. In the absence of spatial validation, it is not possible to determine whether proliferation reflects a small subset of highly proliferative cells or a broader proliferative population. Therefore, proliferation-associated gene expressions in this study are interpreted as a feature of dynamic cellular activity associated with tissue dissociation and culture conditions, rather than as evidence of immaturity or progenitor identity.

The enrichment of organelle- and metabolism-related gene sets in NPCCs-0D cells appears inconsistent with the classical view that stem or progenitor cells are metabolically quiescent and possess limited organelle content [39]. However, this discrepancy may reflect the heterogeneous nature and dynamic state of NPCC preparations immediately following tissue dissociation. Rather than representing a defined progenitor or lineage-specific intermediate, NPCCs‑0D are more appropriately interpreted as a transcriptionally active and adaptive cell population, shaped by enzymatic digestion, disruption of tissue architecture, and exposure to altered environmental conditions. To further examine proliferation-associated features in NPCCs‑0D, we revisited the RNA-seq dataset to evaluate the expression of cell-cycle–related genes. The proliferation marker *MKI67* showed modest enrichment in NPCCs‑0D compared with both NPP and NPCCs‑3D, suggesting the presence of actively cycling cells within this condition. In addition, genes such as *Trefoil factor 2* (*Tff2*), previously associated with epithelial or stress-responsive cell states [40], were also elevated in NPCCs‑0D. Further investigations using higher-resolution approaches, such as single-cell transcriptomics or spatial analysis, will be required to determine whether distinct functional subpopulations exist within NPCC preparations.

On the other hand, the present analysis also confirmed that ECM associated molecular cues are enriched in adaptive porcine pancreatic cells. Changes in ECM–related pathways represent one of the most prominent findings in this study. However, these changes should be interpreted cautiously. The multi-omics datasets generated in this study do not permit discrimination between enhanced ECM synthesis, changes in matrix composition, and alterations in cell–ECM communication. Tissue dissociation during NPCC isolation disrupts native extracellular architecture, and subsequent in vitro culture likely induces adaptive remodeling processes. In addition, differences in cellular composition, including the presence of stromal or epithelial cell subsets, may further contribute to observed ECM-related signatures. Therefore, ECM changes observed in this study are more appropriately interpreted as context-dependent remodeling and adaptation, rather than evidence of differentiation. ECM-related findings highlight dynamic remodeling of the extracellular environment associated with tissue dissociation and culture conditions.

Independent studies have demonstrated that ECM components of the microenvironment play a significant role in multiple pancreatic model systems including mouse endoderm differentiation [41] as well as for human embryonic stem cell-derived β-like cells [42]. Interestingly, by taking advantage of single-cell based mechanistic analysis, it was found that ECM-integrin α5 signaling modulates ductal and endocrinogenesis in bipotent pancreatic progenitors derived from human embryonic stem cells [43]. Strikingly, a very recent study demonstrated that decellularized pancreatic ECM could refine stem cell differentiation microenvironments for organoid development and maturation, verifying the importance of ECM and receptor mediated signals in regulating pancreatic cell fate [44]. In addition to general ECM related factors, the DEGs revealed from current RNA-seq analysis were successfully validated in independent specimens of porcine pancreata and NPCC cells. Previous publications also supported the roles of these molecules in controlling transition/adaptation state in various cell types. For example, biglycan and periostin expressions were positively correlated with cellular differentiation in various mesenchyme-originated progenitor cells [45, 46]. Furthermore, the knockout of HSPA5 impaired progenitor capacity in distal lung epithelial progenitor alveolar type II (AT2) cells [47], while a conditional deletion of HSP90B1 in pancreatic progenitors clearly impaired postnatal pancreatic β cell mass and function, indicating the significance of HSP90B1 in early developmental process [48].

Our transcriptomic and proteomic analyses consistently highlight ECM organization and receptor-mediated signaling pathways as critical determinants of NPCC adaptation. These findings align with prior reports demonstrating integrin signaling in pancreatic progenitors [49] and the role of ECM scaffolds in refining stem cell differentiation [44]. Increased representation of ECM-associated pathways in NPP and NPCCs‑3D highlights the contribution of an intact extracellular niche to cellular homeostasis and adaptive capacity, suggesting that engineering ECM-based culture systems or biomaterials could stabilize NPCC maturation before transplantation. Lastly, we acknowledge that detailed cell composition data (endocrine, acinar, ductal, progenitor-related proportions) were not included in this study. Future work employing single-cell RNA-seq or flow cytometrical analysis could be essential to resolve NPCC heterogeneity. Proteomic findings should be validated by immunostaining or Western blotting of representative proteins. Extending these analyses to human pancreatic progenitors will further clarify the translational relevance of our observations. With these limitations, at this stage, we could consider the differences among NPP, NPCC‑0D, and NPCC‑3D primarily reflect changes in tissue and cell context, disruption of cell-cell and cell-matrix, interactions isolation-induced stress responses and environmental adaptation, rather than a linear developmental hierarchy.

Several limitations and potential solutions in this study are acknowledged. (1) RNA-seq and proteomics analysis was performed with a limited number of biological replicates (*n* = 2–3), which reduces statistical power for dispersion estimation and increases sensitivity to variability and potential outlier effects; (2) Short-term culture is insufficient to drive functional maturation of NPCCs, and the observed molecular changes are more appropriately interpreted as adaptive responses to the in vitro environment rather than progression toward a differentiated state; (3) While overall transcriptional trends distinguish experimental groups, inter-replicate variability is observed, and therefore results should be interpreted at the pathway and functional level rather than relying on individual genes.

## Conclusion

This study provides an integrated transcriptomic and proteomic characterization of neonatal porcine pancreatic tissue and derived cell clusters under different experimental conditions. NPCC samples exhibited substantial molecular changes following isolation and short-term culture, particularly in pathways related to ECM organization, cellular signaling, metabolism, and stress responses. These findings suggest that the observed molecular alterations primarily reflect dynamic, context-dependent adaptations to tissue dissociation, cellular heterogeneity, and the in vitro culture environment, rather than a linear differentiation process. The integration of global transcriptomic profiling, pathway enrichment analyses, and proteomic characterization provides a comprehensive framework for understanding NPCC biology during early post-isolation adaptation. Our results further highlight the importance of ECM remodeling and receptor-mediated signaling pathways in maintaining cellular organization and adaptive responses. These findings suggest that optimization of the extracellular microenvironment may represent a promising strategy to enhance NPCC maturation and functional performance before transplantation.

In summary, this work advances our understanding of the molecular landscape and plasticity of neonatal porcine pancreatic cell clusters and provides a valuable resource for future studies of pancreatic development, xenotransplantation, and cell-based therapies for diabetes. Further investigations using higher-resolution approaches, including single-cell analyses and functional validation studies, will be important to define the specific cellular populations and molecular mechanisms underlying NPCC adaptation and maturation.

## Supplementary Information

Below is the link to the electronic supplementary material.

Supplementary Material 1

Supplementary Material 2

Supplementary Material 3

Supplementary Material 4

Supplementary Material 5

Supplementary Material 6

## Data Availability

All data and methodologies will be made available upon request.
