# Supplementary material for: Integrated transcriptomic and proteomic analysis reveals isolation and culture associated molecular changes in neonatal porcine pancreatic cell clusters
Source: Mol Biol Rep. 2026 Jul 23;53(1):1247. doi: 10.1007/s11033-026-12376-8 (PMC13395948; doi:10.1007/s11033-026-12376-8)
Supplement: Supplementary file 1 — Supplementary Material 1 [file 11033_2026_12376_MOESM1_ESM.pptx]

## Slide 1
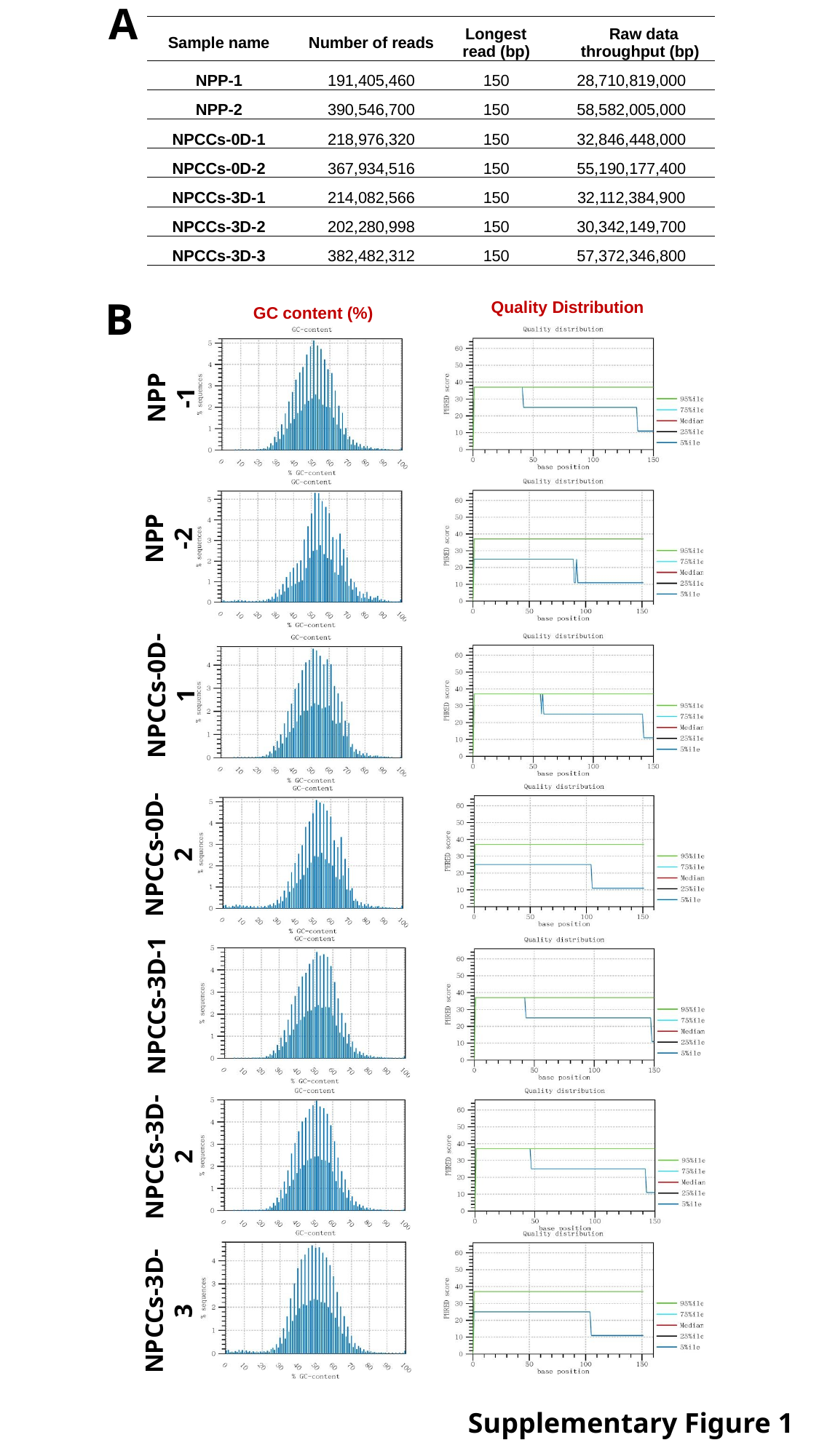

A
| Sample name | Number of reads | Longest read (bp) | Raw data throughput (bp) |
| --- | --- | --- | --- |
| NPP-1 | 191,405,460 | 150 | 28,710,819,000 |
| NPP-2 | 390,546,700 | 150 | 58,582,005,000 |
| NPCCs-0D-1 | 218,976,320 | 150 | 32,846,448,000 |
| NPCCs-0D-2 | 367,934,516 | 150 | 55,190,177,400 |
| NPCCs-3D-1 | 214,082,566 | 150 | 32,112,384,900 |
| NPCCs-3D-2 | 202,280,998 | 150 | 30,342,149,700 |
| NPCCs-3D-3 | 382,482,312 | 150 | 57,372,346,800 |
B
Quality Distribution
GC content (%)
NPP-1
NPP-2
NPCCs-0D-1
NPCCs-0D-2
NPCCs-3D-1
NPCCs-3D-2
NPCCs-3D-3
Supplementary Figure 1

## Slide 2
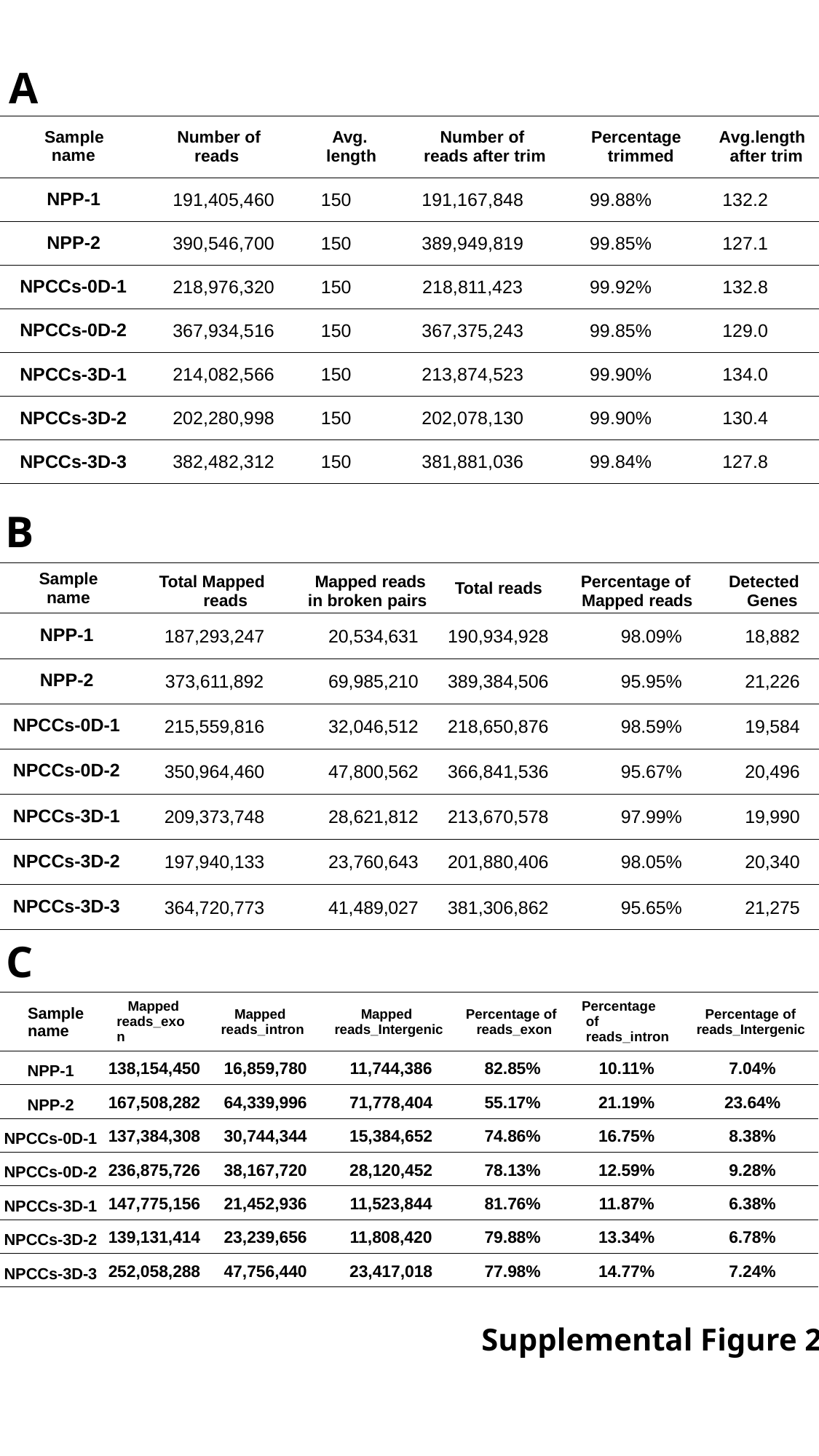

A
| Sample name | Number of reads | Avg. length | Number of reads after trim | Percentage trimmed | Avg.length after trim |
| --- | --- | --- | --- | --- | --- |
| NPP-1 | 191,405,460 | 150 | 191,167,848 | 99.88% | 132.2 |
| NPP-2 | 390,546,700 | 150 | 389,949,819 | 99.85% | 127.1 |
| NPCCs-0D-1 | 218,976,320 | 150 | 218,811,423 | 99.92% | 132.8 |
| NPCCs-0D-2 | 367,934,516 | 150 | 367,375,243 | 99.85% | 129.0 |
| NPCCs-3D-1 | 214,082,566 | 150 | 213,874,523 | 99.90% | 134.0 |
| NPCCs-3D-2 | 202,280,998 | 150 | 202,078,130 | 99.90% | 130.4 |
| NPCCs-3D-3 | 382,482,312 | 150 | 381,881,036 | 99.84% | 127.8 |
B
| Sample name | Total Mapped reads | Mapped reads in broken pairs | Total reads | Percentage of Mapped reads | Detected Genes |
| --- | --- | --- | --- | --- | --- |
| NPP-1 | 187,293,247 | 20,534,631 | 190,934,928 | 98.09% | 18,882 |
| NPP-2 | 373,611,892 | 69,985,210 | 389,384,506 | 95.95% | 21,226 |
| NPCCs-0D-1 | 215,559,816 | 32,046,512 | 218,650,876 | 98.59% | 19,584 |
| NPCCs-0D-2 | 350,964,460 | 47,800,562 | 366,841,536 | 95.67% | 20,496 |
| NPCCs-3D-1 | 209,373,748 | 28,621,812 | 213,670,578 | 97.99% | 19,990 |
| NPCCs-3D-2 | 197,940,133 | 23,760,643 | 201,880,406 | 98.05% | 20,340 |
| NPCCs-3D-3 | 364,720,773 | 41,489,027 | 381,306,862 | 95.65% | 21,275 |
C
| Sample name | Mapped reads\_exon | Mapped reads\_intron | Mapped reads\_Intergenic | Percentage of reads\_exon | Percentage of reads\_intron | Percentage of reads\_Intergenic |
| --- | --- | --- | --- | --- | --- | --- |
| NPP-1 | 138,154,450 | 16,859,780 | 11,744,386 | 82.85% | 10.11% | 7.04% |
| NPP-2 | 167,508,282 | 64,339,996 | 71,778,404 | 55.17% | 21.19% | 23.64% |
| NPCCs-0D-1 | 137,384,308 | 30,744,344 | 15,384,652 | 74.86% | 16.75% | 8.38% |
| NPCCs-0D-2 | 236,875,726 | 38,167,720 | 28,120,452 | 78.13% | 12.59% | 9.28% |
| NPCCs-3D-1 | 147,775,156 | 21,452,936 | 11,523,844 | 81.76% | 11.87% | 6.38% |
| NPCCs-3D-2 | 139,131,414 | 23,239,656 | 11,808,420 | 79.88% | 13.34% | 6.78% |
| NPCCs-3D-3 | 252,058,288 | 47,756,440 | 23,417,018 | 77.98% | 14.77% | 7.24% |
Supplemental Figure 2

## Slide 3
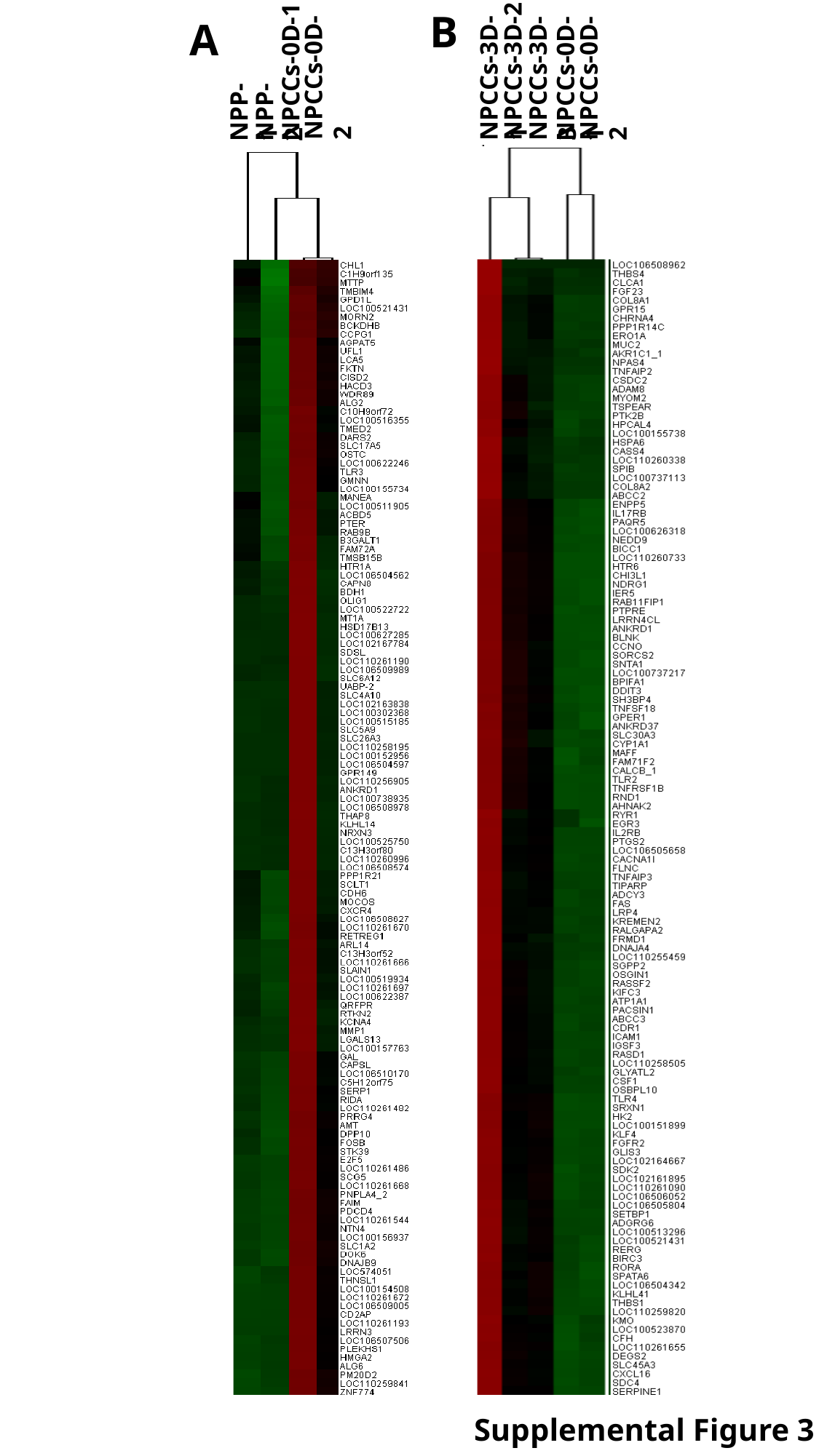

B
NPCCs-3D-2
NPCCs-3D-1
NPCCs-3D-3
NPCCs-0D-2
NPCCs-0D-1
NPCCs-0D-1
NPCCs-0D-2
NPP-1
NPP-2
A
Supplemental Figure 3
