# Supplementary figures and images for: Integrated transcriptomic and proteomic analysis reveals isolation and culture associated molecular changes in neonatal porcine pancreatic cell clusters
Source: Mol Biol Rep. 2026 Jul 23;53(1):1247. doi: 10.1007/s11033-026-12376-8 (PMC13395948; doi:10.1007/s11033-026-12376-8)

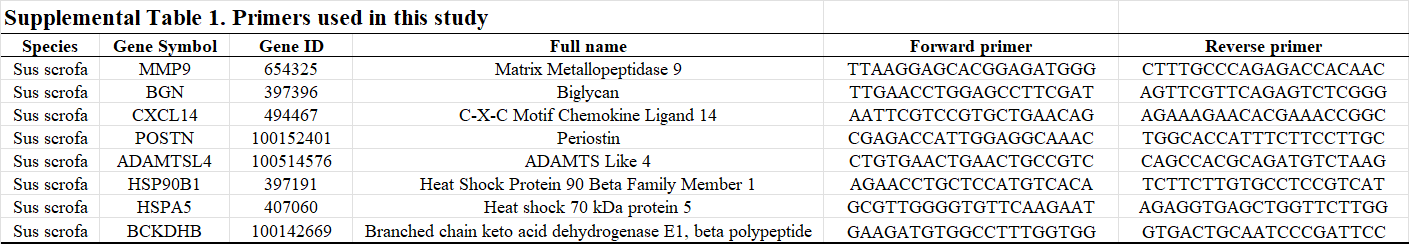

Supplement: Supplementary file 5 — Supplementary Material 5 [file 11033_2026_12376_MOESM5_ESM.tif]
